# Delayed Gastric Emptying after Pancreatic Surgery: Analysis of Factors Determinant for the Short-term Outcome

**DOI:** 10.3389/fsurg.2016.00025

**Published:** 2016-04-25

**Authors:** A. Noorani, Elena Rangelova, M. Del Chiaro, Lars Ragnar Lundell, Christoph Ansorge

**Affiliations:** ^1^Division of Surgery, Department of Clinical Science, Intervention and Technology, Center for Digestive Diseases, Karolinska Institutet, Karolinska University Hospital, Stockholm, Sweden

**Keywords:** pancreatoduodenectomy, pancreatic tail resection, duodenectomy, delayed gastric emptying, risk factors, postoperative pancreatic fistula

## Abstract

**Background:**

Delayed gastric emptying (DGE) frequently complicates pancreatoduodenectomy (PD). Mainly DGE develops as consequence of postoperative intra-abdominal complications (secondary), while the incidence of primary DGE (i.e., not related to surgical complications) has rarely been studied. Moreover, the pathogenesis of DGE is complex and needs to be further elucidated. The present study aimed at highlighting potential mechanisms behind primary and above all secondary DGE by studying a variety of different pancreatic surgical procedures.

**Patients and methods:**

During the time period 2008–2011, 327 patients underwent pancreatic resective procedures at Karolinska University Hospital. Of these, 242 were PD and 56 tail resections, 17 had a duodenal preserving pancreatectomy for chronic pancreatitis, and 15 patients with familial duodenal polyposis had a pancreas preserving duodenectomy. All postoperative courses were assessed and scored according to Clavien–Dindo. The presence of DGE was evaluated and recorded according to the definition launched by the International Study Group for Pancreatic Surgery (ISGPS). Crude associations were studied in a univariate model, followed by a multivariate analysis of the respective factors. The associations were presented as odds ratios (ORs) with 95% confidence intervals (CIs).

**Results:**

In total DGE emerged during the postoperative course in about 40% of the PD cases. About half of those (*n* = 47) were scored as being primary. The majority of the primary DGEs were classified as A (*n* = 26) and only four as grade C, whereas among the secondary cases significantly more patients were scored as grade C (*p* < 0.01). In those submitted to a pancreatic body and tail resection 25% reported DGE. The distribution of the different grades of DGE in patients with a tail resection followed the same pattern with a predominance of Grade A cases with an equal distribution between those being scored as primary and secondary. Duodenal preservation, as well as keeping the pancreas intact following duodenectomy, was not followed by primary DGE. Multivariate risk factor analyses for the development of primary GE revealed no specific risk profile except for high age.

**Conclusion:**

DGE is frequently seen after different surgical procedures directed toward the pancreatic gland. DGE is most commonly seen after PD, and half of these cases are scored as primary DGE. Primary and secondary DGE are seen in one-quarter of the cases even after pancreatic tail resection emphasizing the complex nature of the pathogenesis. Resection of the duodenum as an important mechanism behind DGE is not supported by the present results.

## Introduction

Pancreatoduodenectomy (PD) represents the standard of care for the treatment of pancreatic and periampullary tumors and for pre-cancerous lesions in the head of the pancreas. Even if the pancreatectomy associated mortality today is low, the postoperative morbidity remains high, even in specialized centers (1, 2). The most frequent and hazardous complications are related to the pancreatic remnant, above all in cases of a soft pancreas with a narrow main pancreatic duct, leading to a high incidence of postoperative pancreatic fistula (POPF) and pancreatic stump complications (3–5). The second most frequent complication is delayed gastric emptying (DGE) (6), with an incidence ranging from a few percentages to as much as 80% of the cases. These huge differences in incidence may be depending on which classification applied. Although not life-threatening, and in most cases probably self-limiting, DGE causes significant morbidity due to postponed resumption of oral intake, prolonged hospital stay, and increased costs (7–10). Although DGE is strongly associated with intra-abdominal complications, in particular POPF (11–14), it is also observed in patients without these complications. The latter condition may tentatively be designated as primary DGE, in contrast to the former clinical situation which traditionally has been called secondary DGE. Studies of the pathogenesis of DGE have hitherto not given consistent results, why many aspects on the complex etiology remain unexplored. Moreover, evaluating the underlying mechanisms of DGE after PD is problematic as the procedure itself introduces so many different abnormalities of the gastrointestinal function that it becomes almost impossible to separate the individual components contribution and the possible interaction between those. The PD procedure basically comprises three different components; the removal of the pancreatic head, the duodenum, and the reconstruction gastrointestinal continuity. The latter if of course dependent on the different techniques used (e.g., distal gastrectomy or pylorus preservation) (11, 14–19). In order to apply a stepwise approach to the understanding of the pathogenesis of DGE, the aim of the current study was to compare the incidences of DGE after PD, distal pancreatectomy (DP), duodenum-preserving pancreatic head resections (DPPHR), and pancreas-preserving duodenectomy (PPD). Finally, in this stepwise and hypothesis generating approach, a multivariate analysis was completed to identify factors of specific relevance for the occurrence of primary and secondary DGE.

## Patients and Methods

From the Karolinska Hospitals’ prospective database over pancreatic surgery, all consecutive patients between January 2008 to December 2011 who underwent PD, DP, DPPHR, and PPD were included. All PDs were carried out incorporating a distal gastrectomy, a duct-to-mucosa end-to-side pancreatico-jejunostomy, an end to side hepatico-jejunostomy, a stapled antecolic omega loop gastrojejunostomy, and side-to-side entero-enterostomy. The intraoperative assessments of the pancreatic gland texture and main duct diameter were done according to a standardized protocol (5). In DP, the gland was transected with a stapler above the superior mesenteric vein. In patients with chronic pancreatitis, a DPPHR according to the Berne modification was done (20). The PPD was done as previously described in detail (21). In brief, the duodenal bulb was maintained to which the proximal jejunum was anastomosed end to end or end to side. The main pancreatic duct and common bile duct was inserted end to side to the jejunal loop. Before closure of the laparotomy, abdominal drains were inserted to evacuate abdominal fluid and possible pancreatic juice from the anastomotic or transection areas. The drainage volume and pancreatic amylase concentrations were measured daily. These drains were kept in place at least for 3 days where-after they were withdrawn as indicated by the clinical course of the patient. The postoperative pain management consisted of epidural anesthesia for the first five postoperative days and then oral or intravenous combined treatment with opioids and paracetamol. The postoperative course were assessed and scored according Clavien–Dindo (22). The presence of DGE was recorded and graded according to the definition launched by the International Study Group for Pancreatic Surgery (ISGPS) (6).

### Statistics and Ethics

The statistical analyses were performed using SPSS 19.0 software (SPSS Inc., Chicago, IL, USA). Data were presented as frequencies, mean (±SD) or median and interquartile ranges (IQRs). All tests of statistical significance were two-sided, and a significant difference was considered to occur at an alpha <0.05. Pearson’s chi-square was used to perform significance tests for categorical values as well as the Spearman correlation test. Logistic regression analyses were performed to identify risk factors for DGE, with and without simultaneous adjustment for competing risk factors. Crude associations were studied in a univariate model, which was followed by a multivariate analysis of the respective factors. The associations were presented as odds ratios (ORs) with 95% confidence intervals (CIs). The study protocol had been approved of by the Ethic Committee at Karolinska Institutet, Stockholm, Sweden.

## Results

During the study period, we performed 242 PDs, 56 DPs, 17 DPPHRs, and 15 PPDs. The demographic and disease-specific characteristics of the study population are summarized in Table 1, whereas Table 2 summarizes the postoperative courses and morbidities scored according to Clavien–Dindo (22). Primary DGE was prevailing in cases without any detectable clinical association to a surgical complication. In total, DGE was recorded during the postoperative course in roughly 40% of the PD cases. About half of those (*n* = 47) were scored as primary (Table 3). The distribution, of the DGE grades from A to C, showed that the majority of the primary DGEs were classified as A (*n* = 26) and only four as grade C. A completely different picture emerged among the secondary DGE cases, where significantly more patients were scored as grade C (*p* < 0.01). In patients with DGE classified either as primary or secondary, this complication resulted in a doubling of the hospitalization time.

**Table 1:** **Demographics and clinical characteristics**.

	PD, *n* = 242	DP, *n* = 56	DPPHR, *n* = 17	PPD, *n* = 15
Gender (M/F)	135/107	26/30	13/4	7/8
Mean age (years)	65	62	48	48
Invasive adenocarcinoma	183	17	–	
Neuroendocrine tumor	13	16	–	–
Premalignant/benign	56	23	17 CP	15 FAP
R0 resection rate	36%	32%	na	100%
Type I diabetes	22	5	3	–
Type II diabetes	20	4	3	–
Cardiovascular morbidity	79	20	4	2
Other sign. comorbidity	31	15	2	2

*PD, pancreaticoduodenectomy; DP, distal pancreatectomy; DPPHR, duodenum-preserving pancreatic head resections; PPD, pancreas-preserving duodenectomy*.

**Table 2:** **Postoperative courses and complications scored according to Clavien–Dindo (22)**.

	PD, *n* = 242	DP, *n* = 56	DPPHR, *n* = 17	PPD, *n* = 15
ISPG fistula	44	10	2	4
Grade A	49	8	5	4
B	28	4	0	1
C	22	2	2	1
Clavien grade 1	107	30	9	5
2	51	13	4	5
3a	42	9	2	2
3b	20	4	1	2
4	14	0	0	1
Reoperation	33	2	2	2

*PD, pancreaticoduodenectomy; DP, distal pancreatectomy; DPPHR, duodenum-preserving pancreatic head resections; PPD, pancreas-preserving duodenectomy*.

**Table 3:** **DGE subdivided into primary and secondary in the different patient groups**.

	PD (*n* = 242)	DP (*n* = 56)	PPD (*n* = 15)	DPPHR (*n* = 17)
Primary DGE	47	7	1	2
Grade A	26	5	1	2
B	17	2	0	0
C	4	0	0	0
Secondary DGE	52	7	5	5
Grade A	23	3	3	3
B	11	2	1	0
C	18	2	1	2
Mean post op. hospital stay (days) range	18.5 (5–156)	15.1 (6–77)	16.4 (7–44)	14.6 (6–56)
Mean post op. hospital stay (days) in patients without DGE	13.7	12.3	12.7	9.7
Mean post op. hospital stay (days) in patients with DGE	25.4	23.4	22.0	21.6

*PD, pancreaticoduodenectomy; DP, distal pancreatectomy; DPPHR, duodenum-preserving pancreatic head resections; PPD, pancreas-preserving duodenectomy*.

Patients undergoing PD had slightly higher DGE rates than those submitted to a pancreatic body and tail resection (25%), a difference which did, however, not reach statistical significance. The distribution of the different grades of DGE in DP patients followed the same pattern with a predominance of Grade A cases among those with primary DGE and an equal distribution between those where the DGE was scored as either primary or secondary.

In Table 3 are given the corresponding scorings for patient’s operated on for chronic pancreatitis with head resection and duodenal preservation and also preservation of the gastric anatomy. The same table also details those submitted to a duodenectomy alone. Preserving or resecting the duodenum did not affect the occurrence of secondary DGE. On the other hand, primary DGE was virtually absent except from a few Grade A scorings. As shown in Table 3, DGE again had a significant impact in the length of the postoperative stay.

Univariate analysis of factors associated with the development of postoperative DGE revealed that pancreatic leakage (any grade) emerged as an important determinant factor (Table 4). Associated to this was the surgeon’s assessment of the gland texture and diameter of the main pancreatic duct. However, in the ensuing multivariate analysis, no factor could be identified which exerted a significant impact on the occurrence of any type of DGE (Table 4). The same was true when a separate analysis of primary DGE was completed except for the influence of older age (age 72–84 years, OR; 1.850 CI; 1.017–3.365, *p* = 0.044).

**Table 4:** **Uni (a) and multivariate (b) analysis of risk factors for occurrence of any grade of DGE; in (c) are given corresponding data on primary DGE**.

Variables	*P*-value	OR
**(A) UNIVARIATE ANALYSIS OF RISK FACTORS FOR OCCURRENCE OF ANY GRADE OF DGE**		
Gender	0.222	0.756 (0.483–1.184)
Age (quartiles)	0.237	1.126 (0.925–1.369)
Diabetes	0.161	0.643 (0.348–1.192)
BMI >30	0.555	1.206 (0.648–2.247)
Cardiovascular comorbidity	0.981	0.933 (0.560–1.761)
Respiratory comorbidity	0.145	0.495 (0.192–1.274)
Pancreatic gland risk (5)	0.000	1.705 (1.277–2.276)
Anastomotic leakage risk (5): low risk/high risk	0.001	2.380 (1.411–4.013)
Multivisceral resection	0.517	1.248 (0.638–2.439)
Pancreatic leakage	0.003	2.366 (1.342–4.170)
**(B) MULTIVARIATE ANALYSIS OF RISK FACTORS FOR OCCURRENCE OF ANY GRADE OF DGE**		
Gender	0.323	0.753 (0.429–1.322)
Age (quartile)	0.087	1.252 (0.968–1.620)
Diabetes	0.183	0.586 (0.267–1.286)
BMI ≥30	0.238	1.598 (0.734–3.048)
Cardiovascular comorbidity	0.924	1.037 (0.494–2.175)
Respiratory Comorbidity	0.217	0.502 (0.168–1.499)
Pancreatic gland risk	0.222	1.596 (0.753–3.382)
Anastomotic leakage risk Low risk/High risk	0.876	0.901 (0.244–3.334)
Multivisceral resection	0.871	1.092 (0.378–3.157)
Pancreatic leakage	0.152	1.752 (0.813–3.774)
**(C) MULTIVARIATE ANALYSIS OF RISK FACTORS FOR OCCURRENCE OF PRIMARY DGE**.		
Gender	0.869	0.060 (0.531–2.012)
Age (quartile)	0.104	1.309 (0.946–1.811)
Diabetes	0.603	0.778 (0.302–2.006)
BMI ≥30	0.517	0.687 (0.221–2.138)
Cardiovascular comorbidity	0.483	1.357 (0.578–3.186)
Respiratory comorbidity	0.259	0.414 (0.090–1.915)
Pancreatic gland risk	0.343	0.638 (0.252–1.0616)
Multivisceral resection	0.505	0.589 (0.124–2.791)

## Discussion

The present study confirms and extends previous observations on the frequent occurrence of any kind of DGE in the early postoperative recovery period after pancreatoduodenectomy (7, 17, 23–25). Until now, few large consecutive series of pancreatoduodenectomies have applied the ISPGS consensus definition of DGE (6), and we could therefore conclude that 40% of our patients suffered from DGE and more importantly close to half of these scored their DGE as serious as grade B and C. The strong correlation between postoperative surgical complications after PD and DGE has been addressed in previous publications (4, 6–10). This was confirmed also in our patients, but interestingly, we found that a substantial proportion of these PD patients also suffered from what we defined as primary DGE, i.e., not associated with any detectable surgical complication. These total numbers of primary DGE are higher than previously reported, which again emphasizes the magnitude of the problem but also the relevance of using a prospective data collection process and the strict adherence to the definition of the target complication. In fact, a recent publication (26) comparing three existing definitions of DGE in 55 consecutive patients. These authors were able to show that the incidence of unspecified DGE varied from 6 to 29%, again illustrating the need for objective, universally accepted consensus definitions. Our data also demonstrate the consequences of DGE in the form of prolongation of the postoperative hospital stay. We observed an important difference in the presentation of primary versus secondary DGE, since half of the primary DGE’s were only graded as A and therefore could be considered to be of marginal clinical importance. On the other hand, the secondary DGE’s after pancreatico-duodenectomy were clearly of more severe nature since 18 out of 52 were scored as grade C.

Another important finding of ours was that after a body and tail resection of the pancreas (*n* = 56), DGE was noted in as many as 25% of the cases, by and large equally divided between primary and secondary nature. A substantial proportion of the primary DGE was again graded as A, whereas the secondary ones portrayed a more clinically severe course. The reasons why we included these patients into the study were twofold; first to understand the role played by pancreatic leakage as such on the incidence of DGE. The second reason was to identify the incidence of DGE in pancreatic surgery, when no other physiologic and anatomic changes (27–30) are introduced (such as duodenectomy and/or partial gastrectomy). Even if it can be argued that DGE occurs after a complicated tail resection, it is more unclear why a primary DGE emerges in similar uncomplicated situations. Pancreatic fistula remains, even in cases of body and tail resection, the main cause behind secondary DGE. How much the partial removal of the pancreatic gland, the surgical trauma *per se*, or the role of pain treatment can be involved in the primary DGE remains unclear.

Irrespective of whether pylorus preserving pancreaticduodenectomy or classical Whipple procedures are studied, these operations by definition significantly interferes with important physiological regulatory alimentary canal mechanisms. These might well induce and/or perpetuate DGE, which then operates in conjunction with well-known factors, such as leaking pancreatic juice with or without local inflammatory-infectious reactions (31–35). A wide range of mechanisms have been proposed to cause DGE including the absence of hormonal stimulation elicited by the resection of the duodenum, the denervation/ischemia of the antropyloric region resulting from the interruption of vagal branches and the ligation of the gastric pedicles. The consequences of pylorus preservation on the risk for DGE have not been clearly established. Some studies suggest a higher incidence while others have reported an even lower rate of DGE than after classical Whipple procedures (31–35). We observed that duodenal preserving pancreatic resections in chronic pancreatic cases (n = 17) very seldom were followed by primary DGE. The importance of the pylorus sphincter for the prevention of DGE may also be illustrated by our observation in pylorus preserving duodenectomy. In these patients, we recorded only one case with primary DGE scored as grade A. Otherwise animal experiments suggest the importance of removal of the duodenum, with its content of above all motilin containing endocrine cell (28, 29). Apparently duodenectomy does not have an impact on the development of the DGE in the human setting, as suggested from experiments in canine models.

Another factor which has been suggested to affect the incidence of DGE is the construction of the jejunostomy. An antecolic route is of some importance for minimization of the risk of DGE (25, 36–38), although the theoretical background for this finding remains obscure (39, 40). However, for the sake of standardization all our PD procedures, we constructed an antecolic gastrojejunostomy. All our PD cases had also an entero-entero anastomosis with the ambition to prevent bile reflux into the gastric remnant. The relevance and efficacy of this procedure as well as alternative gastrointestinal reconstructive approaches have to be addressed in future clinical trials.

Delayed gastric emptying has frequently been associated with a number of preoperative risk factors. For instance, preoperative drainage of the bile ducts was associated with a low rate of DGE. Otherwise increased age, cholangitis, pancreatic fibrosis, diabetes mellitus, and malnutrition have been suggested as preoperative factors associated with a risk for DGE after pancreatoduodenectomy (31–35). We were, however, unable to demonstrate that neither of these was of importance for the development of DGE in the early postoperative recovery period neither in a univariate nor in a multivariate model. This was true concerning any kind of DGE. In this context, it has to be born in mind that except for PD and tail resections, the limited sample size prevented a corresponding complete analysis over the impact of the actual procedures as such.

One factor of significance, which needs confirmation, was the impact of old age on the risk for primary DGE. The increasing prevalence of coexisting diseases and medication with age, make studies on healthy individuals beyond the seventh decade of life difficult. It has been reported that gastric emptying of liquids or a mixed meal is delayed in elderly patients (41), while others have observed that age did not alter the fasting and postprandial antral motility, alleged to play an important role in the emptying of solid food. Conversely, fundic activity may be affected by age, which can account for a disturbance in liquid emptying (41).

In conclusion, DGE is frequently seen after different surgical procedures on the pancreatic gland and are often connected with surgical complications. DGE is most commonly seen after PD but about half of the DGE cases are, however, scored as primary DGE. Again primary as well as secondary DGEs are seen in one-quarter of the cases even after pancreatic tail resection emphasizing the complex nature of the pathogenesis. The role of resection of the duodenum for the pathogenesis of primary DGE is not supported by the present results but the preservation of pyloric sphincter seems to be important.

## Author Contributions

AN: study design, data collection, and analysis of data. ER and MC: preparation of the manuscript. LL: study design, analysis of data, and preparation of manuscript. CA: study design, data collection, analysis of data, and preparation of manuscript.

## Conflict of Interest Statement

The authors declare that the research was conducted in the absence of any commercial or financial relationships that could be construed as a potential conflict of interest.
